# Primordial Germ Cell-Like Cells Differentiated *In Vitro* from Skin-Derived Stem Cells

**DOI:** 10.1371/journal.pone.0008263

**Published:** 2009-12-14

**Authors:** Katja Linher, Paul Dyce, Julang Li

**Affiliations:** Department of Animal and Poultry Science, University of Guelph, Guelph, Ontario, Canada; University of Western Ontario, Canada

## Abstract

**Background:**

We have previously demonstrated that stem cells isolated from fetal porcine skin have the potential to form oocyte-like cells (OLCs) *in vitro*. However, primordial germ cells (PGCs), which must also be specified during the stem cell differentiation to give rise to these putative oocytes at more advanced stages of culture, were not systematically characterized. The current study tested the hypothesis that a morphologically distinct population of cells derived from skin stem cells prior to OLC formation corresponds to putative PGCs, which differentiate further into more mature gametes.

**Methodology/Principal Findings:**

When induced to differentiate in an appropriate microenvironment, a subpopulation of morphologically distinct cells, some of which are alkaline phosphatase (AP)-positive, also express *Oct4*, *Fragilis*, *Stella*, *Dazl*, and *Vasa*, which are markers indicative of germ cell formation. A known differentially methylated region (DMR) within the *H19* gene locus, which is demethylated in oocytes after establishment of the maternal imprint, is hypomethylated in PGC-like cells compared to undifferentiated skin-derived stem cells, suggesting that the putative germ cell population undergoes imprint erasure. Additional evidence supporting the germ cell identity of *in vitro*-generated PGC-like cells is that, when labeled with a Dazl-GFP reporter, these cells further differentiate into GFP-positive OLCs.

**Significance:**

The ability to generate germ cell precursors from somatic stem cells may provide an *in vitro* model to study some of the unanswered questions surrounding early germ cell formation.

## Introduction

The process of early gametogenesis requires a succession of coordinated steps, including primordial germ cell (PGC) specification, migration to and colonization of the gonadal ridges, proliferation, and differentiation into more mature gametes [1]. During gastrulation, which occurs at approximately embryonic day (E) 6.25 in the mouse, a population of 4 to 6 germ cell precursors arises in the proximal epiblast in response to inductive signalling from the extraembryonic ectoderm [2]–[6]. At approximately E7.25, a small cluster of 40 to 50 alkaline phosphatase (AP)-positive PGC precursors [7] localizes to the base of the allantois, and this population of cells then migrates into the hindgut of the embryo proper, corresponding to approximately E9.5 in the mouse. Time-lapse analysis has revealed that PGCs then move from the hindgut along the dorsal body wall, eventually colonizing the fetal gonadal ridges by approximately E11.5 to E12.5 [8]. The fate of PGCs that lag behind in the mesentery [8] and ectopic PGCs that accumulate at various sites along the migratory route is unknown.

Investigating how PGCs are set apart as a distinct population and how they further differentiate into mature germ cells may provide important insights into the early events driving gametogenesis, which, despite advances in the field, have remained largely undefined. Studies aimed at elucidating PGC specification have been impeded due to their deeply embedded location in the developing embryo, limited number, and migratory nature [7], [9], [10]. Over the last several years, it has been demonstrated that embryonic stem cells (ESCs) are able to differentiate into gametes [11]–[18]. Several studies have utilized the differentiation of murine ESCs into embryoid bodies (EBs), which were then observed to give rise to putative PGC-like cells [11], [17], [19]. A murine ESC-to-PGC monolayer differentiation has also been described, in which a subpopulation of clusters of cells arising within the first week of differentiation expressed *c-Kit*, while *Vasa* expression was detected during the second week of differentiation [12]. In addition to the derivation of PGCs from murine ESCs, a recent study has reported that cells resembling early germ cells can also be differentiated from human ESCs based on the application of a monolayer culture, and that these cells express *Vasa*, *Oct4*, *Stella*, and *Scp3* [20]. Oocyte-like cells (OLCs), which are representative of more mature gametes, have been produced *in vitro* from somatic stem cells [21], [22]. However, only a few studies conducted with ESCs [19], [20], [23] have focused specifically on characterizing the germ cell precursors that arise in these cultures, and a direct link between PGC-like cells and later-stage germ cells *in vitro* has yet to be conclusively established.

Determining the identity of *in vitro*-generated PGCs has proven difficult given that a significant number of markers originally thought to be “germ cell-specific” have been shown to be expressed in both pluripotent stem cells and early germ cells. For example, *Oct4* is expressed in PGCs and pluripotent cells [24], [25], confounding its use as a means to definitively confirm that putative PGCs arise in a culture in response to *in vitro* differentiation. In addition, the presence of *Mvh* (the murine *Vasa* homolog gene), *Fragilis*, *Stella*, *Dazl*, and *Blimp1* have been detected in ESCs [11], [16], [26], [27]. Use of the SSEA-1 glycolipid antigen for selecting germ cells is also not specific, as it is a marker of ESCs that is also present in various adult tissues [28]. To track the formation of putative PGCs generated from murine ESCs *in vitro*, several studies have applied fluorescent reporters linked to various transcriptional control regions. Such constructs include an Oct4-GFP reporter in which the proximal enhancer was deleted to confer germ cell-specific expression [12], a *Mvh* knock-in GFP reporter [17], and a Stella-GFP reporter [29]. Aside from marker expression, an epigenetic modification unique to migrating PGCs during embryonic development is their ability to undergo imprint erasure [30], [31]. Indeed, this process has been used as a hallmark to study embryonic germ cells [11], [16], [26] and in characterizing PGC-like cells derived from ESCs [19], [23].

In the current investigation, we have characterized PGC-like cells arising from the induced differentiation of skin-derived somatic stem cells, demonstrating through the use of a Dazl-GFP reporter that these putative germ cell precursors are able to give rise to OLCs. Our findings offer an *in vitro* model to specify PGC-like cells in a temporally predictable manner, facilitating investigation of the effect(s) of various factors on their initial formation and providing a means to elucidate the mechanisms governing their further differentiation.

## Materials and Methods

### Isolation and Culture of Undifferentiated Porcine Skin-Derived Stem Cells

Cells were isolated and cultured as previously described [32] from the skin of porcine fetuses collected at day 40 to 45 of gestation (E40-45). Briefly, skin from the back of individual fetuses was cut into 1 to 2 mm^2^ pieces, which were washed three times in Hanks' balanced salt solution (HBSS), digested with 0.2% trypsin for 40 min at 37°C, and treated with 0.1% DNAase for 1 min at room temperature. Tissue pieces were then washed twice with HBSS, three times with DMEM-F12 (1∶1), and mechanically dissociated by vigorous pipetting. The resulting cell suspensions were passed through 40 µm nylon cell strainers (Falcon), centrifuged, and resuspended in stem cell medium [DMEM-F12 (1∶1) containing penicillin/streptomycin supplemented with 1X B-27 (Gibco), 20 ng/ml EGF (Sigma), and 40 ng/ml bFGF (Cell Signaling Technologies)]. Cells were cultured in 100-mm untreated culture dishes (Sarstedt) at 37°C, 5% CO_2_. Non-adherent clusters of individual cells, or “skin spheres”, formed within 48 hr of culture. In order to remove contaminating cells, which attached to the bottom of the culture dishes, cells remaining as spheres in suspension were cultured for 10 days, during which they were passaged twice prior to use. Cells comprising a sphere are referred to as undifferentiated skin stem cells.

### Induced Differentiation

For differentiation experiments, skin spheres were centrifuged, dissociated mechanically into single cells by pipetting, and plated at a final density of 5x10^4^ cells per 60-mm tissue culture dish (Corning) treated with 0.05 mg/ml poly-D-lysine (Sigma) and 0.005 mg/ml laminin (BD Biosciences). Cell were maintained at 37°C, 5% CO_2_ as an adherent monolayer through culture in 0.2 µm-filtered differentiation medium [DMEM, penicillin/streptomycin, 5% heat-inactivated fetal bovine serum (FBS; Invitrogen), 5% porcine follicular fluid, 0.23 mM sodium pyruvate, 0.1 mM non-essential amino acids (Invitrogen), 2 mM L-glutamine (Invitrogen), and 0.1 mM β-mercaptoethanol]. Half the differentiation medium was replaced every 5 days for up to 50 days of culture.

### Alkaline Phosphatase Staining

Cells at day 20 (D20) of induced differentiation were washed with 1X PBS and fixed directly in 60-mm culture dishes in cold 70% ethanol at 4°C for 1 hr, followed by two washes with 1X PBS and incubation for 4 to 6 hr at room temperature in AP staining solution (10 ml: 1 mg sodium α-naphthyl phosphate, 5 mg diazonium salt, 5% borax, and 10% MgCl_2_) [10]. Stained cells were viewed using a Leica DMIL microscope with OpenLab image analysis software (Improvision). The percentage of AP-positive cells in a given 60-mm dish was estimated based on the total cell count at D20 of induced differentiation, the number of PGC-like cells estimated to be present in a dish based on morphological assessment, and the number of PGC-like cells that stained positive for AP.

### Semi-Quantitative Alkaline Phosphatase Enzyme Activity Assay

Passage two skin stem cells or differentiating cells at D20 were washed twice with 1X PBS and resuspended in lysis buffer (1X PBS, 1% Nonidet P-40, 0.5% sodium deoxycholate, and 0.1% SDS supplemented with PMSF and aprotinin). Half of each supernatant was combined with an equal volume of p-Nitrophenyl Phosphate (pNPP) Liquid Substrate System (Sigma) in a 96-well plate and incubated at room temperature for 30 min to 1 hr. Data was plotted as the percent of absorbance at 405 nm corrected for mg of total protein/ml (Bradford assay) relative to undifferentiated skin stem cells.

### RT-PCR or Real Time PCR

Relative mRNA levels were evaluated by real time PCR using cDNA prepared from E40-45 fetal gonadal cells, undifferentiated passage two skin stem cells (D0), PGC-like cells at D20, 25, and 30 of differentiation, or natural porcine oocytes. When possible, reactions were based on an input of 500 ng of total RNA (isolated using the RNeasy kit; Qiagen) and were carried out as described previously [32]. Groups of 15 OLCs collected by mouth-pipetting at D45 to D50 of differentiation or groups of an equivalent number of natural oocytes were transferred into a solution of 14 U of RNase inhibitor (Amersham) and 5 mM DTT (Invitrogen), boiled for 1 min and vortexed for 1 min (repeated three times). Porcine primers used to amplify *Vasa*, *Gdf9b*, *Dazl*, *Gfrα-1*, and *RpII* have been described previously [21], [33], [34]. All other pig-specific forward (FOR) and reverse (REV) primer sequences, annealing temperatures, and product sizes are listed. *Oct4*: FOR 5′-GCCTTTCCCTCGGTGTCT-3′ and REV 5′-CCTTTGTGTTCCCAATTCCTT-3′, 58°C, 182 bp (designed to avoid amplification of *Oct4* pseudogenes; gi:6624722/AJ251914.1); *Fragilis*: FOR 5′-CATGTCGTCTGGTCCCTGT-3′and REV 5′-GTGGAGGCATAGGCCTGG-3′, 60°C, 137 bp (designed based on maximum overlap between human and mouse gene sequences; gi:47682386/BC070243.1 and gi:20086230/AY082484.1, respectively); *Stella*: FOR 5′-TTAATCCAACCCGGACTCAG-3′and REV 5′-TGGTTGAGGTGGATATTCTGG-3′, 60°C, 173 bp (gi:181341661/EW049692.2); *c-Kit*: FOR 5′-TGTATTCACAGAGACTTGGCGG-3′and REV 5′-CGTTTCCTTTGACCACGTAA-3′, 58°C, 124 bp (gi: 31249701/AC141857.2); *Gfp*: FOR 5′-CCTGAAGTTCATCTGCACCA-3′ and REV 5′- GGTCTTGTAGTTGCCGTCGT-3′, 60°C, 196 bp. Cycling conditions were as follows: 95°C-2 min, 40 total cycles of 95°C-15 sec, 56 to 62°C-30 sec (primer-dependent), and 72°C-30 sec and a final cycle of 75°C-30 sec. Parallel reactions were carried out for the porcine *RpII* housekeeping gene to calculate relative mRNA levels by real time PCR using the 2^-[Δ][Δ]Ct^ method [35]. The amplification efficiencies were tested for each primer pair, the specificity of the melt curves was determined, and the integrity of each product was verified by gel electrophoresis (to verify the correct product sizes) and bi-directional sequencing.

### Immunofluorescence

Cells were washed with 1X PBS and fixed in 4% paraformaldehyde overnight at 4°C. After a 10 min incubation in 1X PBS, 0.1% Tween-20, cells were incubated for 20 min in 1X PBS, 1% Triton-X-100 followed by a 4 hr to overnight block in 1X PBS, 5% BSA, 0.05% Triton-X-100 (PBS-B). Incubation with primary antibody, either 1∶500 anti-OCT4 (rabbit; Santa Cruz Biotechnology, H-135), 1∶500 anti-DAZL (mouse; AbCam, ab17224), 1∶500 anti-VASA (rabbit; AbCam, ab13840), 1∶500 anti-c-KIT (mouse; BD Bioscience, 555713), or 1∶500 anti-STELLA (mouse; Chemicon International, MAB4388) was performed overnight at 4°C. The cross-reactivity and specificity of the DAZL antibody has been verified previously in our laboratory [34], and the VASA antibody is listed by the manufacturer to cross-react with the pig (see on-line antibody specification sheet). The antibodies against OCT4 and c-KIT were tested by Western blotting using porcine extracts, verifying the presence of proteins migrating at the expected molecular weights. Cells were washed in PBS-B, incubated with a 1∶500 dilution of the appropriate secondary antibody (anti-mouse or anti-rabbit FITC- or PE-conjugated) for 1 hr at room temperature, and washed with PBS-B. Negative controls were included in which cells were incubated in the presence of secondary antibody only to verify specificity of staining. Prior to mounting in fluorescent mount medium (DakoCytomation), cells were incubated with 1 µg/ml Hoechst for 25 min and washed three times with PBS-B. Images were obtained using an Olympus BX-UCB microscope with MetaMorph image analysis software (Universal Imaging Corporation).

### Sodium Bisulfite Genomic DNA Sequencing

Genomic DNA was isolated from denuded porcine oocytes, fetal skin, passage two skin stem cells, passage five skin stem cells that had been *in vitro* cultured in an undifferentiated state for 25 days, and D25 PGC-like cells using a method based on lysis with proteinase K. Following an EcoRI digest, 300 ng of denatured DNA was modified with sodium bisulfite as described [36] using sodium bisulfite at a final concentration of 4M and hydroquinone at 1 mM. A 260 bp sequence spanning the porcine *H19* DMR1 was amplified using primary and nested PCR primers [36]. Each 50 µl reaction included either 5 µl of bisulfite-treated genomic DNA (primary PCR) or 2 µl of primary product (nested PCR), 3.8 µl of each 100 µM primer, FastStart Taq DNA polymerase (Roche), dNTPs, 10X FastStart buffer without MgCl_2_, and MgCl_2_ at a final concentration of 5 mM. Primary and nested PCRs were performed using established cycling parameters [36], and products were cloned into pGEM-T Easy. Individual clones were sequenced, and cytosine to uracil conversions were identified by sequence alignment.

### Cloning of the Lentiviral Gene Transfer Plasmid pL-SIN-Dazl-GFP

The self-inactivating lentiviral gene transfer plasmid (pL-SIN-Lenti-EF1α-GFP) was a kind gift from Dr. James Ellis (Hospital for Sick Children/University of Toronto, Toronto, Canada) [37]. In order to substitute the *EF1α* constitutive promoter with the full-length porcine *Dazl* promoter sequence (from −1981 to +14 bp) cloned from oocyte cDNA and characterized previously [34], pL-SIN-Lenti-EF1α-GFP was cut partially with BamHI/NcoI. A multiple cloning site (BamHI, SalI, MluI, PstI, XhoI, NheI, NcoI) was introduced into the linearized vector, resulting in pL-SIN-Lenti-MCS-GFP. The full-length *Dazl* promoter was excised from the pGL3 vector backbone with SmaI/HindIII and subcloned into the pBluescriptII-KS intermediate cloning vector, introducing BamHI and XhoI sites flanking the promoter sequence. The promoter fragment was then transferred into a BamHI/XhoI cut pL-SIN-Lenti-MCS-GFP vector, resulting in the lentiviral gene transfer plasmid pL-SIN-Lenti-Dazl-GFP.

### Production of Recombinant Lentiviral Particles

293FT cells seeded at 4×10^6^ cells per 10-cm dish one day prior to use were co-transfected with 3.6 µg of lentiviral gene transfer plasmid (either pL-SIN-Lenti-EF1α-GFP or pL-SIN-Lenti-Dazl-GFP), 2.4 µg of gag-pol expression plasmid (HPV-275), 2.4 µg of rev expression plasmid (p633), 2.4 µg of tat expression plasmid (HPV-17) and 1.2 µg of VSV-G envelope plasmid (pHCMV-VSV-G) using Lipofectamine 2000 (Invitrogen) following the manufacturer's recommendations. After 24 hr at 37°C, the media was changed. Virus-containing supernatant was collected from the cells at 48 and 72 hr post-transfection and passed through 0.45 µm sterile syringe filters. Viral particles were concentrated by ultracentrifugation at 28,500 rpm for 2 hr at 4°C and resuspended in an appropriate volume of TBS+MgCl_2_ buffer. Functionality of viral particles and an estimated titer were determined by transducing 293FT cells with serial dilutions of pL-SIN-Lenti-EF1α-GFP viral stock. The percentage of live GFP-positive cells relative to the total number of infected cells 72 hr post-transduction was analyzed by flow cytometry (FACS Calibur; Becton Dickinson).

### Lentiviral Transduction of PGC-Like Cells

Approximately 1.25×10^5^ of non-adherent PGC-like cells collected at D25 of induced differentiation were resuspended in 1 ml of a 1∶1 mixture of conditioned medium and fresh differentiation medium. Polybrene at 8 µg/ml and Dazl-GFP lentivirus at an MOI of approximately 10 were added to the cells, followed by incubation for 4 hr at 37°C. Transduced cells were centrifuged, washed with differentiation medium, and reseeded onto the original monolayers maintained in 60-mm culture dishes in differentiation medium at 37°C. After 5 to 14 days, cells were evaluated for GFP expression.

### Statistical Analyses

The mean difference in relative AP activity is represented as the percent of undifferentiated skin-derived stem cells. Mean fold changes in mRNA levels were calculated relative to D30 PGC-like cells. For each set of results, independent experiments were repeated at least three times, with data representing the mean±SEM of all repeats within an individual experiment. Data were analyzed by t-test or one-way analysis of variance (ANOVA) followed by the Tukey test for multiple comparisons to determined statistical differences between groups (denoted by a star or different letters, respectively) using GraphPad Prism analysis software. Results were considered significant at P<0.05.

## Results

### PGC-Like Cells Morphologically Resemble Natural PGCs

**Figure 1:**
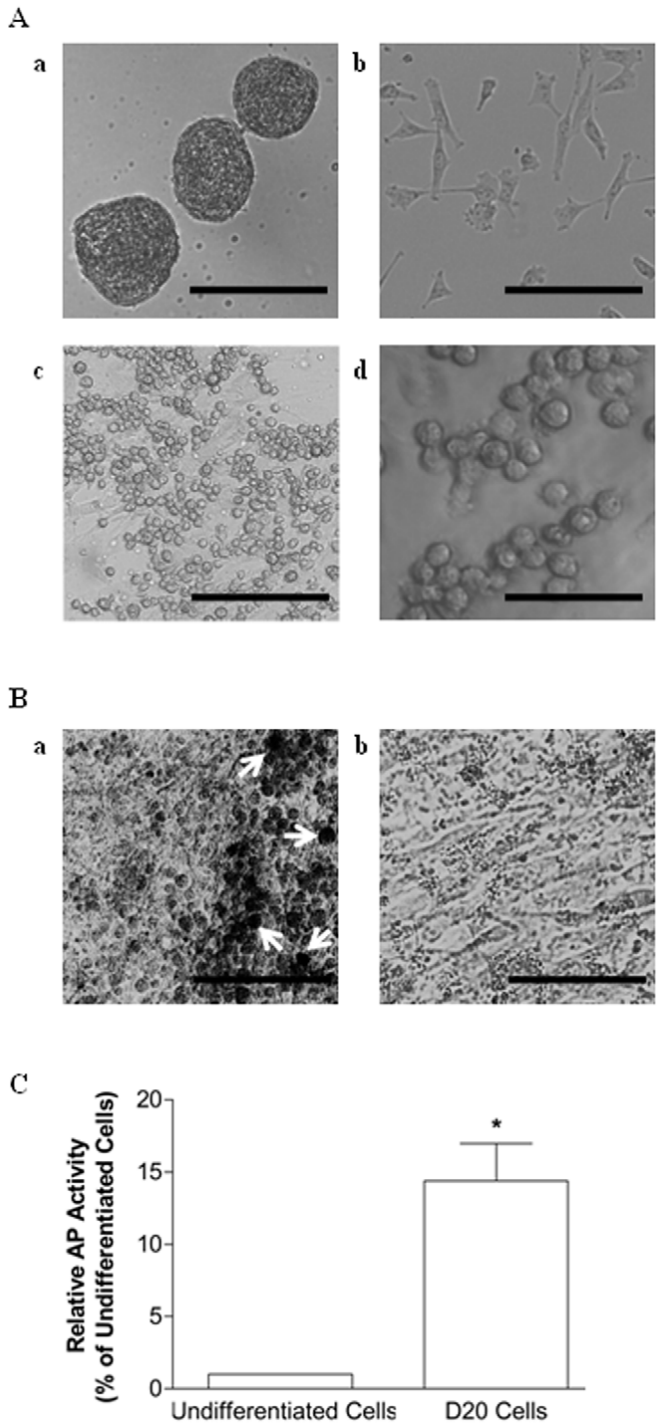
Morphology and AP activity of PGC-like cells differentiated from porcine skin-derived stem cells. (A) Bright field images of (a) undifferentiated skin-derived stem cells maintained as spheres at passage 2 (100X) or (b) dissociated passage 2 skin stem cells after being plated in differentiation media for 24 hr in 60-mm dishes (100X). Loosely adherent or non-adherent putative PGCs at D20 of differentiation (c, d) could be distinguished from the somatic monolayer by their large size, round shape, and blebbing at (c) 100X and (d) 200X, respectively. (B) D20 differentiating cells, including a confluent monolayer with loosely adherent PGC-like cells, were fixed and stained for AP. Approximately 0.25% of the large, round PGC-like cells were AP-positive (a) compared to background levels associated with (b) the supporting somatic cell layer (100X). Size bar = 100 µm (Ad), 200 µm (Aa,b,c and B). (C) AP activity in undifferentiated skin-derived stem cells compared to D20 differentiating cells. A * denotes a statistical difference between the two groups (P<0.05). Absorbance values were normalized against total protein (mg/ml) and expressed as a percent relative to undifferentiated cells.

Undifferentiated skin stem cells within non-adherent spheres (Figure 1A, a) were dissociated, and within 24 hr of plating in differentiation medium, the stem cells were sparsely attached to the 60-mm culture dishes, adopting a “fibroblast-like” morphology (Figure 1A, b). Over a two-week period, the adherent differentiating cells proliferated to form a confluent layer of cells. Between days 15 to 30 of induced differentiation, morphologically distinct round cells appeared in the cultures. Representative images of these cells at D20 of differentiation are shown in Figure 1A, c and d. These cells, which ranged in diameter from approximately 15 to 20 µm, appeared either as single cells with a slightly blebbed contour or formed loosely adherent clusters resting on top of the existing monolayer of “fibroblast-like” cells. In contrast, the size of individual dissociated, undifferentiated skin stem cells was approximately 5 µm in diameter (data not shown). A sub-population of the round cells gradually detached and grew as a separate non-adherent population of single and clustered cells. This morphology is consistent with the typically oval or round shape, irregular or “blebbed” contour, and large nucleus associated with porcine PGCs dissociated from gonadal ridges [38] or murine PGCs [39], which are known to be larger in size compared to somatic cells. Some of the adherent, morphologically distinct PGC-like cells at D20 of differentiation stained positive for AP (Figure 1B, a), while the underlying monolayer was largely AP-negative (Figure 1B, b). The number of morphologically distinct cells that appeared in the cultures at this time point was approximately 1×10^4^ per 60-mm dish, which is approximately 1.4% of the total cell population (7×10^5^ cells) undergoing differentiation. The percentage of AP-positive PGC-like cells within a given 60-mm dish was estimated to be approximately 0.25% of the total cell population. The AP staining shown here is similar to results presented elsewhere for natural porcine PGCs [38], [40]. In addition, an enzyme activity assay indicated that AP levels were approximately 15-fold higher (P<0.05) in cells undergoing differentiation than levels detected in undifferentiated skin stem cells (Figure 1C).

### PGC-Like Cells Express Markers Relevant to Germ Cell Formation

**Figure 2:**
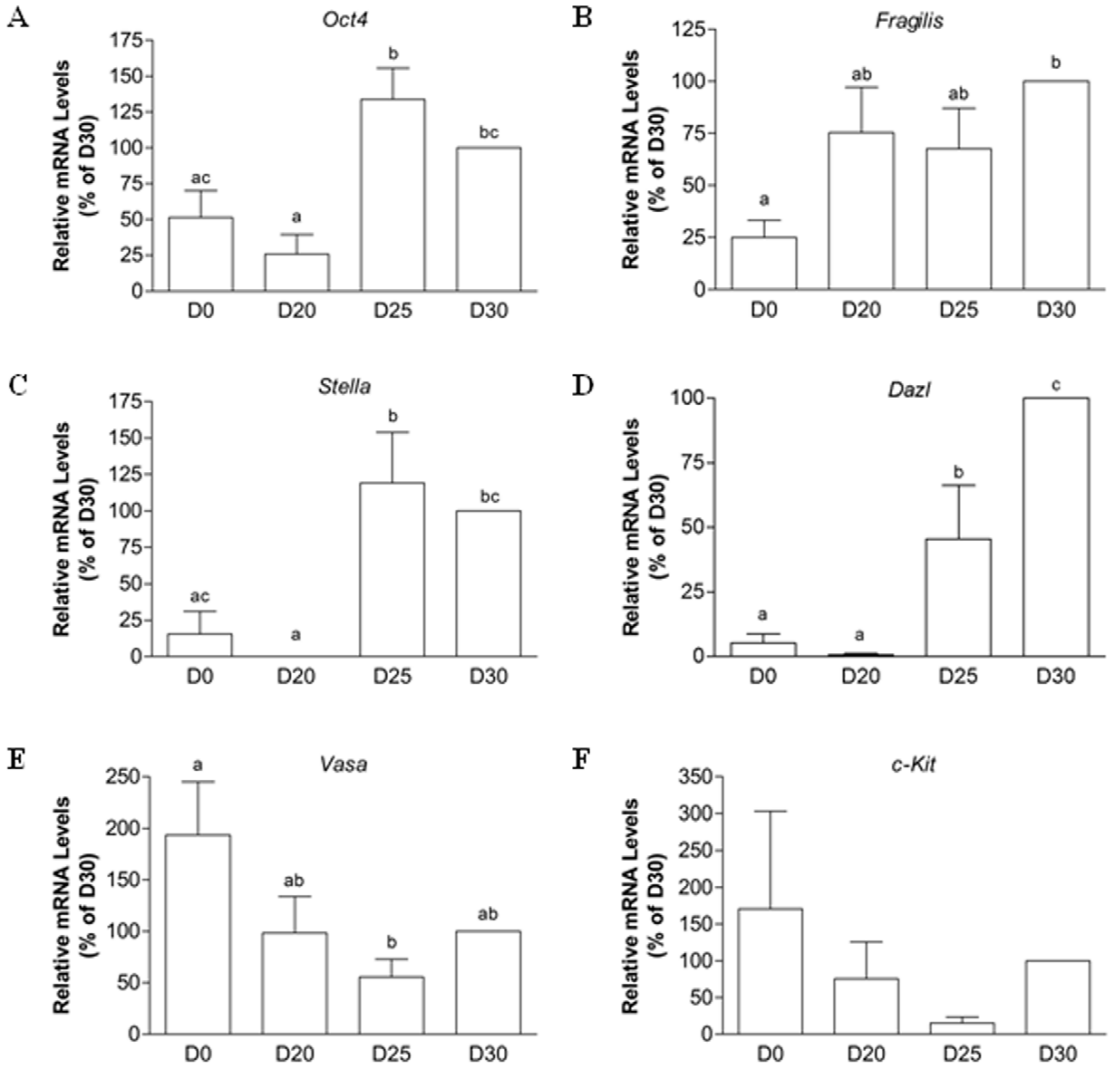
The detection of germ marker mRNA in PGC-like cells. The expression of several marker genes known to be associated with PGCs was determined by real time RT-PCR. Relative quantification of (A) *Oct4*, (B) *Fragilis*, (C) *Stella*, (D) *Dazl*, (E) *Vasa*, and (F) *c-Kit* mRNA levels in D0 undifferentiated skin-derived stem cells and D20, D25, and D30 non-adherent PGC-like cells. Levels were normalized for the *RpII* housekeeper, and results are presented relative to D30 PGC-like cells. Data represent the mean±SEM of four independent experiments, with different letter subscripts denoting statistical differences between groups (P<0.05).

**Figure 3:**
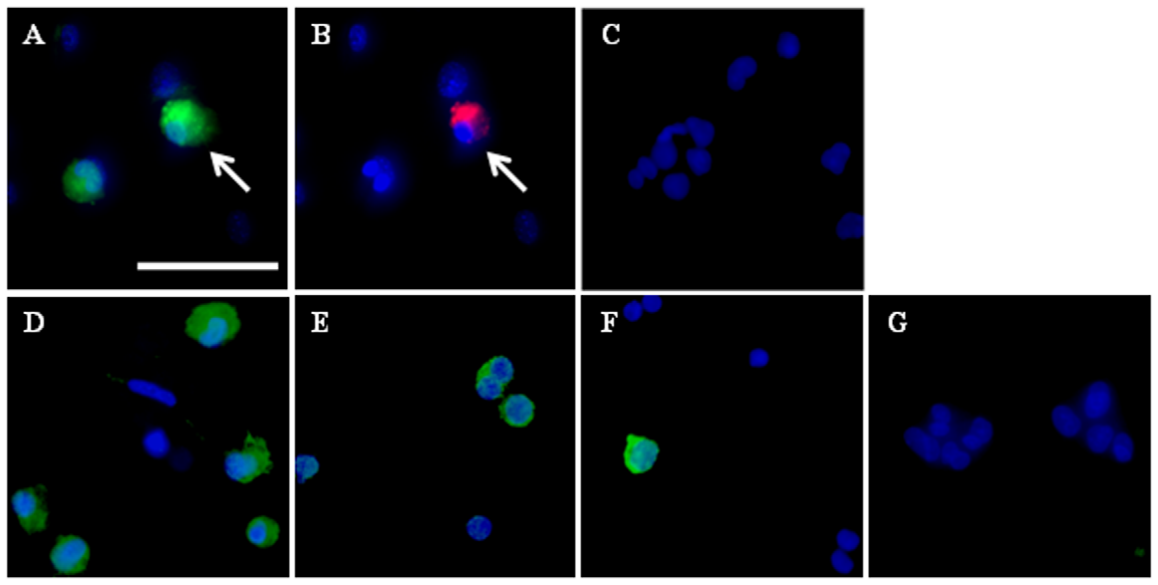
Detection of germ markers in PGC-like cells. Immunolocalization of A) OCT4, B) VASA, D) STELLA, E) c-KIT, and F) DAZL in D30 PGC-like cells. White arrows indicate cells co-staining for OCT4 and VASA (A and B). Controls in which cells were probed with secondary antibody alone (either anti-rabbit or anti-mouse-FITC/PE) were negative (C and G, respectively). Counter-staining with Hoechst was conducted to detect nuclei. Panels are shown at 400X magnification (bar = 50 µm).

To examine whether the subpopulation of putative early germ cells identified based on morphology also expressed marker genes known to be associated with PGCs, RT-PCR was performed. The specificity of each amplified product in PGC-like cells was verified by size on agarose gels against the migration of bands detected in fetal gonadal cells (results not shown) and by bi-directional sequence analysis. Once the specificity of the products was established, temporal changes in relative mRNA levels for each of the markers were evaluated by semi-quantitative real time PCR (Figure 2A-F) using the porcine *RpII* housekeeping gene as a normalizer. Efficiency testing against *RpII* revealed equal amplification for each of the target genes across a template dilution series, as plots of the log of serial dilutions of cDNA versus Ct values obtained for the housekeeper and each target gene exhibited comparable slopes, and the delta Ct versus log of serial dilutions plots resulted in a straight line with a slope less than 0.1. The Ct values for all products were below 35. Transcripts for most of the markers could be detected in undifferentiated skin stem cells, a result which varied depending on the fetal skin isolation from which D0 cells were obtained. The expression of germ markers at the undifferentiated stage was not surprising given recent reports that many “germ cell-specific” genes are also expressed in pluripotent stem cells [13], [16], [26], [27]. Nevertheless, there were certain dynamic changes in mRNA levels between undifferentiated skin stem cells at D0 and PGC-like cells collected at various time points during induced differentiation. *Oct4* mRNA levels increased by approximately 3-fold (P<0.01) in D30 PGC-like cells compared either to those collected at D20 or in undifferentiated stem cells (Figure 2A). Although not statistically significant, *Oct4* expression appeared to be lower in D30 PGC-like cells compared to levels obtained at D25. *Fragilis* mRNA levels were higher (4-fold, P<0.01) at D30 in PGC-like cells compared to undifferentiated stem cells (Figure 2B). Expressed before induced differentiation, the *Stella* transcript was almost undetectable at D20, while its expression was dramatically up-regulated by D25 of differentiation (P<0.01; Figure 2C). *Dazl* expression was very low to undetectable in undifferentiated stem cells, reaching peak levels in D30 PGC-like cells. An approximate 20-fold induction (P<0.0001) in *Dazl* mRNA levels was observed between D0 and D30 (Figure 2D). Although *Vasa* was expressed in undifferentiated stem cells, there was nevertheless a dynamic change in its expression profile during induced differentiation, with lowest levels observed at D25 (Figure 2E). While no statistical differences were observed in *c-Kit* expression, changes in mRNA levels followed a pattern similar to the temporal profile established for *Vasa* (Figure 2F). It should be noted that, with the exception of *Fragilis*, relative mRNA levels for all the other marker genes analyzed were in the order of 10 to 33-fold higher in gonadal cells than in PGC-like cells (data not shown). To confirm the expression of germ cell markers at the protein level, immunofluorescent analyses were also performed. As shown in Figure 3, OCT4, VASA, STELLA, KIT, and DAZL were detected in PGC-like cells collected at D30 of induced differentiation (Figure 3A, B, D, E, and F, respectively), with some of the cells co-staining for OCT4 and VASA (Figure 3A&B). This time point was chosen to assess the presence of each protein, as all of the germ markers were also expressed at the mRNA level at this stage in the differentiation culture. No signal could be detected in negative controls probed with anti-rabbit (Figure 3C) or anti-mouse (Figure 3G) secondary antibody only.

### PGC-Like Cells Undergo Imprint Erasure

**Figure 4:**
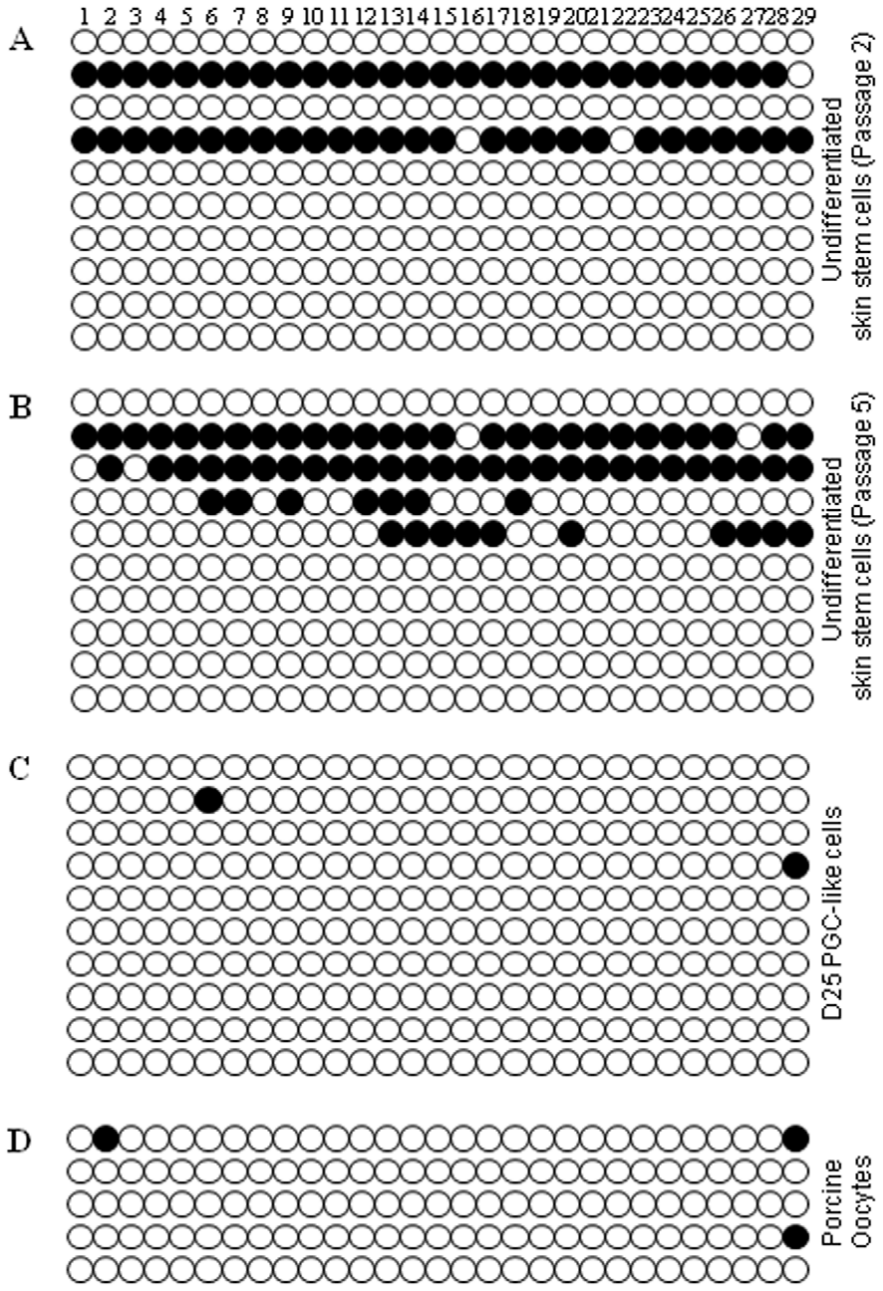
The DNA methylation profile in DMR1 of the porcine *H19* gene locus. Sodium bisulfite sequencing revealed that the percentage of unmethylated CpGs in (A) undifferentiated skin stem cells at passage 2, (B) undifferentiated skin stem cells at passage 5 (cultured for 25 days in the absence of differentiation media), and (C) D25 PGC-like cells was 81%, 76%, and 99%, respectively. (D) Porcine oocytes served as a control for the expected maternal imprint (98% unmethylated). Open and black circles represent unmethylyated and methylated cytosines, respectively.

It has been demonstrated that epigenetic imprints are established during gametogenesis in a parent-specific manner [41]–[43], and that the DNA methylation status of imprinted genes is erased and reset during PGC development [44], [45]. Sodium bisulfite sequencing was therefore performed to analyze the methylation status within DMR1 of the *H19* 5′ flanking region, which is part of the *Igf2-H19* gene locus known to regulate monoallelic expression of these two imprinted genes (*H19* is maternally inherited, while *Igf2* is expressed by the paternal allele) and which has been used to assess epigenetic imprints established during fetal development and gametogenesis [46], [47]. As shown in Figure 4A, 19% of the 29 individual CpG sites were methylated in undifferentiated skin stem cells at passage 2, which was similar to the methylation profile determined for fetal skin (21% methylated; results not shown). In marked contrast, by D25 of differentiation, 99% of CpG sites were unmethylated in DMR1 (Figure 4C). As a positive control, 98% of the CpG sites were found to be unmethylated in porcine oocytes (Figure 4D), which is in agreement with the 90% unmethylated status reported previously for the pig *H19* DMR1 in oocytes [36]. The demethylation observed in the PGC-like cells was specific to induced differentiation, as culture of skin stem cells in an undifferentiated state for a similar period of time (25 days, 5 passages) did not alter the methylation profile, with 24% of the sites found to be methylated (Figure 4B). These results suggest that imprint erasure occurred in the D25 PGC-like cells undergoing induced differentiation.

### PGC-Like Cells Are Able to Further Differentiate into Oocyte-Like Cells

**Figure 5:**
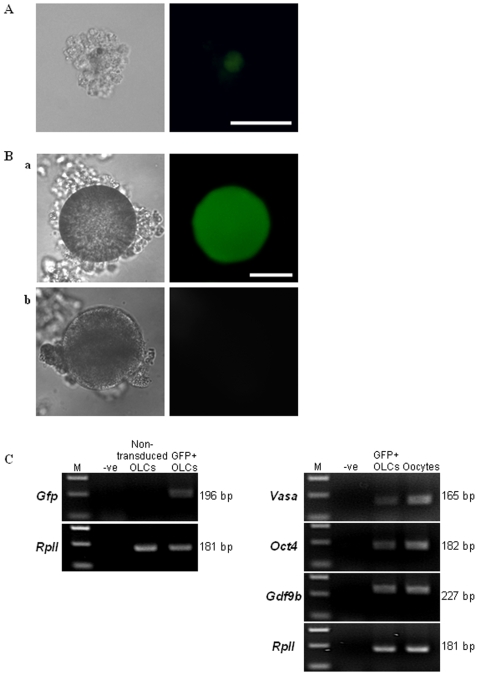
PGC-like cells lentivirally transduced with the Dazl-GFP reporter give rise to OLCs. Bright field and fluorescent images of a (A) representative aggregate and (B) an OLC derived from D25 PGC-like cells transduced with a Dazl-GFP lentivirus. (A) A GFP+ putative germ cell within an aggregate. bar = 100 µm. (B) After further differentiation, a GFP+ OLC was obtained (a, upper panel). OLCs generated from non-transduced PGC-like cells were GFP- (b, lower panel). bar = 50 µm. (C) Representative agarose gel images depicting the presence of *Gfp* mRNA in transduced OLCs (upper panel on the left). GFP+ OLCs also expressed *Vasa*, *Oct4*, and *Gdf9b* (panels on the right), confirming their germ cell identity. M: DNA marker, -ve: a reaction without RT enzyme.

A lentiviral approach was employed to investigate whether the characterized PGC-like cells were indeed germ cell precursors that possessed the intrinsic machinery required for further differentiation into OLCs. A recombinant viral vector was generated by replacing the *EF1α* constitutive promoter with the germ cell-specific porcine *Dazl* promoter characterized in our laboratory [34] to drive expression of the GFP reporter gene. Upon further culture of lentivirally transduced D25 PGC-like cells, ranging from 5 to 15 days, either aggregates of cells or OLCs developed much like those described previously [21]. In the aggregates, which generally formed prior to OLCs, a single, larger (approximately 35 µm in diameter) Dazl-GFP-positive cell was surrounded by other smaller, non-GFP-expressing cells (Figure 5A). The absence of a GFP signal in the surrounding support cells demonstrated the specificity of the porcine *Dazl* promoter in directing expression of the fluorescent reporter to germ cells. The process of generating aggregates was inefficient, as only up to 10 GFP-positive aggregates were present in any given 60-mm dish into which the transduced PGC-like cells were seeded following incubation with lentivirus. After further differentiation (up to D50), a small number of GFP-positive OLCs (varying from 0 to 10) spontaneously appeared in the cultures, with a diameter close to 100 µm in size (Figure 5B, a). No GFP signal could be detected in a non-transduced negative control (Figure 5B, b). RT-PCR performed on cDNA that was isolated from groups of OLCs selected under a fluorescent microscope by mouth-pipetting further confirmed that *Gfp* mRNA was specifically present in OLCs arising from Dazl-GFP lentivirally transduced PGC-like cells (Figure 5C, top panel). These same manually selected late-stage GFP-positive OLCs also expressed the germ markers *Vasa*, *Oct4*, and *Gdf9b* (Figure 5C, bottom panel), which is consistent with what we have reported previously [21] as well as with the expression of these genes in natural porcine oocytes.

## Discussion

While it has been shown that cells resembling PGCs can be differentiated from ESCs *in vitro*, to our knowledge, the current investigation is the first to characterize PGC-like cells generated from the induced differentiation of somatic-derived stem cells. We characterized putative germ cell precursors, demonstrating their close resemblance to PGCs based on (1) morphology and (2) marker expression profiles at the mRNA and protein levels. Furthermore, these putative PGCs (3) underwent imprint erasure and (4) were able to give rise to OLCs, demonstrating that this morphologically distinct population of cells corresponds to PGCs arising in culture.

Germ cell development requires a series of multiple well-orchestrated steps which involve the concurrent up- and down-regulation of specific markers. In our PGC-like cell population, which morphologically resembled germ cell precursors, a small percentage also exhibited AP activity, which is associated with PGC specification and the earliest stages of their migration. The population of putative germ cells that arose *in vitro* was by no means representative of the initial cluster of cells that arise *in vivo* in the developing embryo, as events in the culture dish are not necessarily synchronized. It is likely that some of the AP-negative PGC-like cells had already progressed past the “specification” stage and were more representative of the later migratory stage, during which AP may no longer be optimally detected. *Oct4*, a key regulator of the pluripotent phenotype, is first expressed in the inner cell mass of the blastocyst, but is subsequently down-regulated and eventually only expressed in germ cells [48]. Both *Oct4* mRNA and its protein could be detected in the morphologically distinct PGC-like cells. *Oct4* expression appeared to be dynamically controlled, as its mRNA was present in undifferentiated skin stem cells, but levels were enriched in PGC-like cells. It has been reported that human *Oct4* is highly expressed in migrating PGCs, but is silenced once PGCs enter the gonadal ridges and continue to differentiate into more mature gametes [49]. Interestingly, *Oct4* expression appeared to drop off at later stages of induced differentiation.

The expression of other germ cell marker genes also underwent dynamic changes during differentiation. *Fragilis* is thought to initiate the repression of homeobox genes in early germ cell precursors [50] and is also expressed in ESCs, suggesting a role in maintaining pluripotency [4]. The *in vitro* expression profile determined for *Fragilis* revealed that its mRNA was enriched by D20, a stage in the differentiation at which PGC-like cells first appear as a morphologically distinct population. Increased *Fragilis* expression was sustained at later stages, suggesting that these cells are not homogenous and may represent putative PGCs at various stages of specification/differentiation. *Stella*, which is also associated with the maintenance of pluripotentcy in ESCs, has been shown to be up-regulated in murine PGCs by E7.25 and may specifically mark cells committed to the germ line, thereby segregating them from a somatic cell fate [4]. Its expression is maintained in migrating germ cells until E13.5 to E15.5, corresponding to post-colonization of the murine fetal gonads [4]. Interestingly, the temporal expression profile for *Stella* in the *in vitro*-generated PGC-like cells was similar to *Oct4*. Lack of *Stella* expression at the earlier stage could reflect a transition of cells committing to the germ lineage. *Dazl* is essential for PGC development, as knock-out mice lack a germ cell population [51]. Its expression at the mRNA level was low or undetectable in undifferentiated skin stem cells and early-stage PGC-like cells, but DAZL protein was present in some later-stage PGC-like cells. As peak *Dazl* mRNA levels were not observed until the later stage of culture, it is possible that in order for *Dazl* to be optimally expressed, the induction of other key germ cell determinants would first need to occur. *Vasa* is expressed in PGCs after colonization of the gonadal ridges and in developing germ cells up until post-meiotic stages [52], and human VASA protein is most abundant in mature oocytes [53]. The murine VASA homologue (MVH) protein is also highly expressed in ESCs [13], [16], [26], which corresponds with our finding that *Vasa* mRNA levels were high in undifferentiated skin stem cells. Its expression appeared to be down-regulated during the early stages of induced differentiation, reflecting a possible transition to a stage at which germ cell specification was initiated. c-KIT, a tyrosine-kinase receptor that binds stem cell factor (SCF), is expressed in PGCs during their migration to the gonadal ridges [54], and the trend toward increased *c-kit* mRNA levels in PGC-like cells at later stages of culture may reflect a potential transition in the progression of *in vitro* gametogenesis. It is clear that PGC-like cells at any given time point during *in vitro* differentiation are by no means a uniform cell population, and that germ cells at various stages of specification/differentiation may be represented by the morphologically distinct cells characterized here.

The striking similarities reported between expression profiles established for pluripotent stem cells and PGCs raise interesting questions regarding the spatial and temporal origin of the two cell types and their fate. Early germ cell precursors arising in the proximal epiblast of the developing embryo are a highly mobile population [8]. It is therefore possible that, prior to colonizing the gonadal ridges, specified PGCs may not be entirely committed to the germ lineage, instead representing a migrating group of pluripotent cells. Indeed, their migratory nature may provide a means for some of these cells to establish niches in non-gonadal somatic tissues. This notion is in accord with a recent hypothesis proposing that *Oct4*-positive very small embryonic-like stem cells (VSELs) are deposited in somatic tissues during development and sustained as a small quiescent population during adult life [55], playing important roles in tissue/organ regeneration [56]-[59]. We found that cells isolated from porcine fetal skin express *Oct4* (unpublished results), and it is therefore tempting to speculate that these pluripotent cells could descend from a primitive stem cell/putative PGC population originally seeded in the dorsal body wall during the early stages of PGC migration. The inhibitory somatic environment maintained in the skin could potentially keep these cells in a dormant state, and their pluripotent/germ cell potential would only become apparent when placed in an appropriate microenvironment such as the *in vitro* differentiation culture.

The *in vitro*-generated PGC-like cells were further characterized by investigating their ability to undergo imprint erasure by assessing changes in DNA methylation. The somatic status of imprinted genes is progressively erased during PGC migration through the hindgut and dorsal mesentery *in vivo* [30], [44], [60], and in the mouse, imprinted genes are hypomethylated in germ cells by E13.5 [61], [62]. In porcine PGCs, the methylated status of DMRs in the maternally inherited *Igf2-H19* gene locus decreases from E24 to 28, and in female PGCs, these regions are completely demethylated by E30 [45]. Compared to DNA isolated directly from fetal porcine skin or from undifferentiated skin stem cells, the DNA from D25 PGC-like cells was found to be hypomethylated within DMR1 of the *H19* gene locus. The reduced percentage of methylated CpGs observed in D25 PGC-like cells was likely due to imprint erasure and not merely a response to the time spent in culture, as undifferentiated skin stem cells maintained for 25 days *in vitro* did not exhibit this epigenetic change. While the bisulfite sequencing results presented here for cultured skin-derived stem cells do not reflect the 50% CpG methylation status (1∶1 ratio) associated with monoallelic *H19* gene expression indicative of the expected maternal imprint, similar results were reported in another recent study. Analysis of the methylation status of an analogous DMR in the bovine *Igf2-H19* gene locus performed on a panel of tissues derived from several near full-term bovine fetuses revealed differential methylation of CpG sites at ratios other than 1∶1 [63]. For example, in bovine brain, kidney, lung, and liver, the percentage of methylated CpGs ranged from 21–44%, 27–36%, 13–31%, and 36–50%, respectively [63]. Changes in the expected ratio also have been reported elsewhere in both cattle and mice [42], [64]. The reason for the observed deviations is unclear, and possibly could be attributed to the heterogeneity of cell types present in a specific sample.

Lentiviral-mediated delivery of the Dazl-GFP reporter [34] into D25 putative germ cells demonstrated that the PGC-like cells characterized here give rise to OLCs. Cells at this stage of differentiation were chosen for transduction based on their marker expression profile, which indicated a possible commitment to the germ lineage. While we have shown previously that estradiol- and progesterone-secreting aggregates that morphologically resemble follicle-like structures are generated in the induced differentiation culture [21], to our knowledge, this is the first study linking PGC-like cells and OLCs *in vitro*. At the onset of folliculogenesis, female porcine PGCs (or primary oocytes at this later stage of development) increase in size up to a diameter of 27 µM [65]. Following lentiviral transduction, GFP was expressed in the growing putative oocytes, which averaged approximately 30 to 35 µm in size and were located at the center of aggregates. GFP was also highly expressed in OLCs, which reached a size of approximately 100 µm in diameter. We have previously characterized *in vitro*-generated OLCs, demonstrating that they morphologically resemble oocytes and express markers associated with mature germ cells such as *Oct4*, *Vasa*, and *Gdf9b* [21]. The novelty of the current investigation was the finding that GFP-positive OLCs, which expressed these markers in a manner consistent with our previous report, were generated from PGC-like cells labeled with Dazl-GFP, establishing a link between PGC-like cells and OLCs *in vitro*.

Identifying how PGCs are specified and further differentiate may help to eluciate the causes of infertility and other reproductive disorders that are initiated during embryonic development. In addition, producing oocytes *in vitro* would be a major advance in developing a model to study gametogenesis. Murine ESCs can be differentiated into PGCs, mature germ cells, and blastocysts [66]. Our study demonstrates that somatic stem cells also have the developmental capacity to sustain the differentiation of cells closely resembling PGCs under suitable *in vitro* conditions.
